# Genetic diversity analysis of Peking gecko (*Gekko swinhonis*) in mid-Eastern China based on mitochondrial COI and Cyt *b* gene sequences

**DOI:** 10.1080/23802359.2019.1623724

**Published:** 2019-07-10

**Authors:** Cheng-He Sun, Da-Wei Liu, Ya-Lin Huang, Yong-Wu Zhou, Sen-Lin Hou, Chang-Hu Lu

**Affiliations:** a College of Biology and the Environment, Nanjing Forestry University, Nanjing, China;; b Forest Police Identification Center of National Forestry Administration, Nanjing Forest Police College, Nanjing, China

**Keywords:** Genetic diversity, mitochondrial DNA, Peking gecko

## Abstract

To understand the genetic diversity of Peking gecko (*Gekko swinhonis*) populations in its endemic region, 60 individuals were sampled from Lushan, Qi, and Linying counties in Henan Province, China. Through PCR amplification and Sanger sequencing, 120 sequences with lengths of 652 bp (COI) and 739 bp (Cyt *b*) were obtained, and nine haplotypes were detected for each gene. Overall, results indicated that Peking gecko populations in China have high genetic diversity and significant genetic differentiation. This study provides necessary scientific basis for the protection of Peking gecko germplasm resources.

## Introduction

The Pecking gecko (*Gekko swinhonis*) is a reptile within the family Gekkonidae. It feeds mainly on small insects and lives in crevices of buildings, trees, and rocks. This species is unique in China, and, in 2010, it was listed as vulnerable in the International Union for Conservation of Nature red list of endangered species. *Gekko swinhonis* is nocturnal, good at climbing, and, when attacked, it can break its tail to escape. It is traditionally used in medicine and to catch a variety of insects (Li and Zhou 2007; Yan et al. 2010). At present, researches on *G. swinhonis* mainly focus on its reproductive ecology and medicinal use, and research on population structure have mostly been based on morphological characters, with molecular markers technology rarely being used (Heinicke et al. 2012).

Mitochondrial DNA (mtDNA) is an ideal material for solving molecular evolution and phylogenetic issues. Compared with nuclear genes, mitochondrial genes have a simpler structure and lower molecular weight, faster evolutionary rate, and no tissue specificity. Therefore, mitochondrial genes are widely used in vertebrates’ phylogeny, biogeography, and conservation genetics studies (Boore 1999; Birky 2001; Doucet-Beaupré et al. 2010). Among mitochondrial genes, the evolutionary rates of cytochrome oxidase I (COI) and cytochrome *b* (Cyt *b*) genes are suitable for the detection of population level differences, and have been widely used to assess the genetic variation and phylogeny of several animal groups (Weibel and Moore 2002; Lian-Wei et al. 2012; Laopichienpong et al. 2016). In the present study, 60 Peking gecko individuals from three different geographical groups in China were selected as the research objects. Their COI and Cyt *b* genes were amplified and sequenced, and the populations were analyzed for genetic diversity to provide a theoretical basis for the conservation of this species.

## Materials and methods

**Figure 1.**
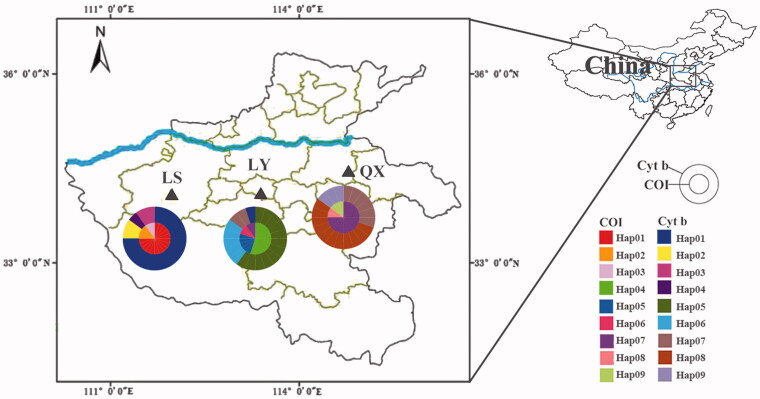
Distribution of sampling locations and haplotypes.

The Peking gecko individuals were collected from Lushan County (LS), Qi County (QX), and Linying County (LY), all in Henan Province, China, from August to September 2018 (Figure 1). The tail muscle was sampled from 20 individuals at each geographical location and stored at –20 °C for later use. After sampling, the specimens were stored at the Museum of Nanjing Forestry University, Nanjing, China.

Muscle samples were rinsed in distilled water, dried, and shredded. DNAiso Reagent (Beijing Baori Medical Biotechnology Co., Ltd., Beijing, China) was used to extract DNA, which was eluted with 50 μL EB buffer and stored at –20 °C. Specific primers for COI (F: 5′-TAAAGACATTGGCACCCTCT-3′, R: 5′-TTCGTGGGTGTGGTATGGTG-3′) and Cyt *b* (F: 5′-CTCGAAAATCCCACCCAGTAC-3′, R: 5′-GAGGATTAGTGCTATGACGCC-3′) genes were designed and synthesized by Nanjing Tsingke Biotechnology Co., Ltd. The two PCR reactions were performed in a total volume of 30 μL, including 15 μL 2 × PCR Premix Taq^TM^, 0.9 μL upstream and downstream primers, 4 μL DNA template, and 9.2 μL double-distilled water. The optimized PCR reaction profile was as follows: pre-denaturation at 95 °C for 5 min; 35 cycles of denaturation at 95 °C for 30 s, annealing at 55 °C for 30 s, and extension at 72 °C for 1.5 min; further extension at 72 °C for 10 min. The amplicons were then detected in 1.5% agarose gels, and those of the correct size were excised and sent to Nanjing Tsingke Biotechnology Co., Ltd. for purification and sequencing.

Sequences were analyzed in SeqMan (Swindell and Plasterer 1997) and manually edited. Genetic diversity parameters, including haplotype number, haplotype diversity index (Hd), nucleotide diversity (pi), and average nucleotide differences (K), were calculated in DNAsp 5.10.01 software (Rozas 2009). MEGA 7.0.26 was used to calculate the genetic distance between populations (Kumar et al. 1994). The genetic differentiation index *F*_st_ and the analysis of molecular variance (AMOVA) were both calculated in Arlequin 3.5 (Ben Dhia and Rateau 2005).

## Results

Using SeqMan, 120 sequences with 652 bp (COI) and 739 bp (Cyt *b*) were analyzed and compared. The average percentages of T, C, A, and G nucleotides were 32.7%, 24.8%, 24.8%, and 17.7%, respectively, in COI sequences and 29.1%, 27.7%, 28.5%, and 14.6%, respectively, in Cyt *b* sequences. In both gene sequences, the content of A + T was higher than that of G + C.

There were 105 variable sites (accounting for 16.1% of the total number of sites) in the COI sequences of the three populations, and these included three single-variable sites and 102 parsimony-informative sites. The COI sequences obtained encoded 217 amino acids and defined nine haplotypes. There were 140 variable sites (accounting for 18.9% of the total number of sites) in the Cyt *b* sequences of the three populations, all corresponding to parsimony-informative sites. The Cyt *b* sequences obtained encoded 246 amino acids and also defined nine haplotypes. The distribution of haplotypes is shown in Figure 1. In COI, Hd, pi, and K were 0.801, 0.0614, and 40.051, respectively, and in Cyt *b* these were 0.840, 0.0727, and 53.722, respectively.

The average genetic distance among the three populations was analyzed based on the two gene sequences. The average genetic distances between LS and QX, LS and LY, and QX and LY were 0.127, 0.135, and 0.135 for COI, and 0.145, 0.143, and 0.035 for Cyt *b*, respectively. Overall, LS and LY were the most genetically distant populations. The AMOVA results are displayed in Table 1. The Fst of COI and Cyt *b* genes was 0.83355 and 0.74915, respectively, with *p* = 0.00. The results of the *F*st test showed that there were significant differences among the three populations (*p* < 0.001). The inheritance among populations accounted for 83.35% and 74.91% of the total genetic variation, suggesting that most of the population variation in Peking gecko was due to genetic variation among populations.

**Table 1.** AMOVA analysis of *G. swinhonis* populations based on COI and Cyt *b* gene fragments.

Source of variation	d.f.		Sum of square		Variance components		Percentage of variation (%)	
	COI	Cyt *b*	COI	Cyt *b*	COI	Cyt *b*	COI	Cyt *b*
Among populations	2	2	933.976	1212.823	25.25541 Va	29.82130 Va	83.35	74.91
Within populations	52	57	262.254	569.184	5.04335 Vb	9.98569 Vb	16.65	25.09
Total	54	59	1196.23	1782.008	30.29876			
Fixation Index			COI *F*_st_=0.83355			Cyt b *F*_st_=0.74915		

## Discussion

Previous studies have shown that Peking geckos are morphologically different among areas, but morphological data alone cannot fully reflect the phylogenetic relationships between groups. Combining morphological and molecular data can better explain biological evolution and potential related factors. In the absence of background data pairs, multiple sets of sequence data of different gene regions are used to obtain more objective information.

The number of individuals sampled from each geographic location in the present study was larger than that sampled by Yan et al. (2010), but fewer samples were collected. Zardoya and Meyer (1996) found that among the 13 protein-coding genes of vertebrate mtDNA, COI, and Cyt *b* genes contained the best phylogenetic information. The evolutionary rate of COI is relatively slow, which is suitable for the analysis of closely related taxa, and the evolutionary rate of Cyt *b* is moderate, which is suitable for the analysis of intraspecific to interspecific genetic information. Therefore, at present many scholars combine COI and Cyt *b* information to analyze genetic differences within and between species. Here, 652-bp COI and 739-bp Cyt *b* gene sequences were obtained for three Peking gecko populations. The content of A + T was higher than that of G + C, in agreement with the heterogeneity of the distribution of the four nucleotides in the mitochondrial genome (Lian-Wei et al. 2012; Webb and Moore 2005; Pereira et al. 2011).

Folmer et al. (1994) considered that an increase in A + T content at the third position provides obvious evolutionary advantages for mtDNA genes. The nucleotide diversity index can clearly reveal the degree of mtDNA polymorphism in a population. In the present study, the nucleotide diversity indices of LS and LY populations were higher than 0.01, and their haplotype diversity and average nucleotide differences were also high, indicating a higher level of genetic diversity than that found in QX. The AMOVA revealed that individuals from LS were genetically different from that of LY and QX, suggesting that the genetic differentiation between these populations is high and that gene exchange is low.

In summary, using mitochondrial COI and Cyt *b* genes as analytical tools, the genetic diversity and differentiation of Peking gecko individuals from three geographical locations were studied. The results showed that there is significant genetic differentiation among the three populations, and that their genetic diversity is high. Due to the sampling constraints, this study does not fully reflect the population differentiation and genetic diversity of Pecking gecko in China. However, results indicate that human factors have had little influence on the diversity of this species. The present results also provide scientific basis for the protection of Peking gecko germplasm resources and basic information for further research on the evolution of this species.
